# Eye disease and international travel: a critical literature review and practical recommendations

**DOI:** 10.1093/jtm/taad068

**Published:** 2023-05-16

**Authors:** Jay Jun Lee, Mark T Forristal, Fiona Harney, Gerard T Flaherty

**Affiliations:** Department of Ophthalmology, University Hospital Galway, Galway, Ireland; Department of Ophthalmology, Temple Street Children’s University Hospital, Dublin, Ireland; Department of Ophthalmology, University Hospital Galway, Galway, Ireland; Department of Ophthalmology, University Hospital Galway, Galway, Ireland; School of Medicine, University of Galway, Galway, Ireland; School of Medicine, University of Galway, Galway, Ireland; School of Medicine, International Medical University, Kuala Lumpur, Malaysia

**Keywords:** High altitude headache, keratitis, contact lenses, high altitude retinopathy, eye injuries, surgical tourism

## Abstract

**Rationale for review:**

Eye diseases pose a significant public health and economic burden, particularly for travellers exposed to ocular hazards who may lack access to specialist eye care. This article offers an evidence-based review for travel-health practitioners, with a particular emphasis on ocular infections and trauma that are more prevalent among travellers. Providing an overview of these issues will allow travel health practitioners to comprehensively address ophthalmic considerations of travel.

**Methods:**

A systematic literature search was conducted on PubMed and Embase electronic databases, using keywords related to travel medicine and ophthalmology. Inclusion was based on the relevant contribution to epidemiology, aetiology, diagnostics, management and long-term consequences of travel-related eye conditions. The data were analysed using narrative synthesis.

**Key findings:**

This literature review highlighted that various travel-related eye conditions may occur. Travellers should be aware of the risk of travel-related ocular complications, which can arise from ocular infections, high-risk activities, high altitude and space travel. The economic and logistical challenges associated with medical tourism for ophthalmic procedures are discussed. For travellers with pre-existing eye conditions or visual impairment, careful planning may be needed to promote eye health and ensure safety of travel.

**Conclusions:**

Travel medicine practitioners should have a comprehensive understanding of the major ocular risks associated with overseas travel, including eye infections, eye injuries and solar eye damage. Further research in this area can enhance overall wellness and alleviate the burden of ocular diseases on travellers. Evidence-based guidelines based on research can also improve the quality of care and prevent long-term vision problems.

## Introduction

Travel medicine is a highly dynamic specialty that focuses on pre-travel preventative care. Travel exposes individuals to a wide range of ocular hazards while also impacting access to specialist eyecare. Eye diseases and visual impairment have a significant public health impact and economic burden. As global tourism returns to pre-pandemic levels, travel medicine practitioners should be knowledgeable about the ocular complications that may arise during international travel. According to the United Nations World Tourism Organisation projections for 2023, international tourist arrivals could recover to between 80 and 95% of pre-pandemic levels, highlighting the importance of informed travel health advice.^1^ Ophthalmic considerations for travel are a relatively under-investigated topic, especially considering the growth of global tourism.^2^

This article offers an evidence-based review for travel-health practitioners, with a particular emphasis on ocular infections and trauma that are more prevalent among travellers. The review will explore a variety of ocular conditions that can arise or worsen in different environments, including high altitude and space travel. This information aims to allow more informed advice to potential travellers after a comprehensive risk assessment, to reduce the frequency of ocular complications of travel and exacerbation of pre-existing eye conditions.

## Methods

Manual searches of PubMed and Embase electronic databases were conducted, in February 2023, with combinations of the following keywords: ‘ophthalmology’, ‘travel medicine’, ‘travel-related eye infections’, ‘space travel’, ‘high altitude’, ‘travel-related eye injuries’, ‘ophthalmic tourism’, ‘air travel’, ‘contact lens use’, ‘visual impairment’ and ‘teleophthalmology’. The search strategy also included a grey literature search which accessed relevant guidelines. Where abstracts were available in English, non-English language articles were also consulted. The reference lists of articles were screened for additional sources not yielded by the primary literature search. Two authors assessed abstracts and identified relevant titles independently. Included texts were reviewed in full, screened for relevance and assessed based on the Oxford Centre for Evidence-Based Medicine levels of evidence.^3^ Inclusion was based on their relevance to epidemiology, aetiology, diagnostics, management and long-term consequences of travel-related eye conditions. The subject areas listed in the Body of Knowledge of the International Society of Travel Medicine (https://www.istm.org/bodyofknowledge2) were used as a reference to ensure that there was broad coverage of the subject.

## Results

### Air travel and eye conditions

Air travel can greatly affect the ocular surface due to the low humidity inside airplanes, resulting in dryness and discomfort with symptoms such as itching, severe pain, headaches, eye watering and contact lens intolerance. In addition, the lower atmospheric pressure on airplanes can cause reduced oxygen tension, leading to ischaemic changes in the nerve and retina. Individuals who have undergone ocular surgery with gas instillation are particularly at risk as lower atmospheric pressure can affect intraocular gas.^4–9^

Ocular pain is reported in 1–2% of air travellers and is classified as an airplane headache (AHA).^10^ Diagnostic criteria for AHA include at least 2 attacks of severe pain during airplane travel lasting less than 30 minutes, with both episodes occurring unilaterally in the fronto-orbital region, with associated jabbing, stabbing or pulsating pain, with no accompanying symptoms and not attributable to other disorders.^11^ AHA tends to occur in landing or descent but may occur during take-off. AHA has a male predominance and tends to occur between the ages of 25 and 30. These headaches are believed to be secondary to sinus barotrauma or vasodilatation in cerebral arteries due to changes in cabin pressure. The fronto-orbital pain is explained by barotrauma to trigeminal nerve endings in the ethmoidal sinuses.^11–13^ Analgesics, non-steroidal anti-inflammatory agents and triptans have been shown to be effective in prophylaxis. Compression of the pain region, the Valsalva manoeuvre, extension of the earlobe, chewing and yawning have also been found to provide relief of symptoms.^12–15^

Ischaemic optic neuropathy, exacerbation of diabetic cystoid macular oedema and neuro-ocular vestibular dysfunction (NOVD) have all been associated with air travel.^16–18^ Air turbulence can cause NOVD or motion sickness. Air travel is not recommended for 2–6 weeks post intraocular sulphur hexafluoride (SF6) or perfluoropropane (C3F8) instillation until the gas has been fully absorbed as the gases can expand in lower atmospheric pressure conditions, causing a rapid rise in intraocular pressure.^19^

Air travel does not pose a problem for individuals with glaucoma as the controlled atmospheric pressure of the airplane cabin compensates for the decrease in pressure at high altitudes that would otherwise increase intraocular pressure ^20^ However, the dim lighting conditions in airplane cabins can cause mydriasis and increase the risk of pupillary block, which can lead to acute angle close glaucoma, an ocular emergency.^21^

### Travel to high altitude destinations

High altitude represents a natural stress due to low oxygen partial pressure and barometric pressure. In normal response to hypobaric hypoxia, the retinal vessels become more engorged and the optic disc more hyperaemic.^22^ High altitude retinopathy (HAR) is a spectrum of pathological changes that occurs in an individual who is exposed to a hypobaric hypoxic environment. They can be seen in most trekkers who ascend beyond 4900 m above sea level. The retinopathy produces retinal oedema, haemorrhages, ischaemia and optic disc swelling.^23^ The degree of severity depends on the length and intensity of exposure, the rate of environmental change, as well as patient factors such as dehydration, oxygen consumption and underlying cardiac or respiratory conditions.^22^^,^^24^^,^^25^ HAR is often detected incidentally as it is usually asymptomatic. Most cases resolve spontaneously and do not require intervention.^26^ In a small subset of patients who develop HAR that results in blurring of vision or loss of visual field, the principles of management are to increase oxygen perfusion with supplemental oxygen and descend to a lower altitude. Besides retinopathy, the development of non-arteritic ischaemic optic neuropathy (NAION) has also been described with travel to high altitudes.^27^

Prophylactic medications, such as acetazolamide, can be effective, but individuals should be aware of potential side effects like a transient myopic shift and have proper corrective measures in place.^29^ High altitude illness, including acute mountain sickness, is primarily prevented by proper acclimatization, gradual ascent, staying hydrated and avoiding alcohol and smoking.^28^

### Space travel

With the increasing number of civilians travelling to space, there are growing concerns about health risks associated with space flight. One such risk is a novel entity termed Space-Flight Related Neuro Ocular Syndrome (SANS). SANS consists of a group of clinical features resulting from exposure to prolonged microgravity. Due to it being a relatively new condition, the underlying pathophysiology is still being debated. The two leading hypotheses are either a cephalad shift or compartmentalization of cerebrospinal fluid.^30^ Common findings after a space mission include hyperopic shift, cotton wool spots and optic disc swelling. In a case series, lumbar punctures in astronauts post-space flight missions with optic disc oedema were as high as 28.5 cm H_2_O.^31^ While no clear management strategy has been recommended, oral acetazolamide has been shown to decrease CSF pressure, but signs of SANS may persist for years after returning to terrestrial conditions.^32^^,^^33^ It is expected the frequency of SANS will rise as more people engage in commercial space travel.

### Visual impairment and travel

Visual impairment encompasses a broad spectrum of conditions that affect a person’s ability to see. It refers to any degree of vision loss, from mild to severe, that cannot be corrected with glasses or contact lenses. Travelling can pose significant challenges for individuals with visual impairment. These challenges can range from difficulty navigating unfamiliar surroundings to a reduced ability to fully experience and appreciate new environments. Navigating new environments can be one of the biggest challenges for individuals with visual impairment. A cane can be a helpful tool that provides stability and enables people to detect obstacles and changes in elevation. Other tools and techniques, such as guide dogs, tactile paving and audio cues, can also help people navigate their surroundings.

To avoid animal quarantine when travelling with a service dog, patients should be advised to plan well in advance. It is important to research the specific requirements for each country and prepare all necessary documentation in advance. In general, most countries require canine health and rabies records, microchip documentation, a vet-issued health certificate, a letter from a healthcare professional stating the need for the service dog and proper identification gear like a vest or harness. The visually impaired traveller should contact the destination country’s embassy or consulate for breed restrictions and appropriate government agencies for animal quarantine policies. It is also advisable to translate all documents into the native language of the visiting country to ensure compliance with their legal requirements.

When travelling, individuals with visual impairment may require extra time and planning to find transportation, locate accommodation and navigate unfamiliar streets and landmarks. Consulting with a healthcare provider or mobility specialist can help determine the best strategies for safe and comfortable travel.^34^ Unfortunately, individuals with visual impairments may be more susceptible to safety risks and crimes. The US Department of Justice reports that people with visual impairment are four times more likely to be victims of crimes such as robbery and assault.^35^ Limited ability to assess their environment, recognize potential danger and respond to threatening situations can increase the risk of victimization for travellers with visual impairments.

In addition to the challenges of navigating new environments, individuals with visual impairment may also have difficulty fully experiencing and appreciating new surroundings. Visually impaired individuals may miss out on visual details and cues that help them understand and appreciate new environments, which can limit their enjoyment of travel experiences. This, in turn, can lead to significant stress and anxiety, which can further impede their ability to fully engage with their surroundings. The psychological impact of visual impairment on travellers is therefore an important consideration, and efforts should be made to optimize the travel experience for individuals with visual impairment.

To address these challenges, it is important to provide support and resources for individuals with visual impairment who are travelling. This may include access to specialized travel agencies that can provide tailored support, such as audio guides for sightseeing and accommodations that cater to the needs of visually impaired individuals. Additionally, travel companions, family members or friends can provide emotional support and assistance with navigating unfamiliar surroundings (Table 1).

### Traumatic eye injury and adventure activities

Eye injuries can be subclassified as closed globe and open globe injuries.^36^ The mechanism of injury may be due to coup, countercoup and anteroposterior compression or horizontal expansion of the tissue.^37^ High-velocity impacts can result in globe rupture secondary to the rapid increase in intraocular pressure and subsequent scleral rupture. Adventure travel presents a significant risk of traumatic eye injury. Bungee jumping is associated with ocular injury via a sudden increase in venous pressure from the rapid acceleration and deceleration forces involved. This can result in intraocular haemorrhages, retinal detachments and direct traumatic injury from the bungee cord itself.^38^^,^^39^

Barotrauma related to scuba diving can lead to eye injury via mask squeeze. Mask squeeze occurs due to a pressure difference between the inside of the mask and the surrounding water. This pressure difference increases as the diver descends leading to a ‘suction’ over the area where the mask is applied. The suction pressure can lead to conjunctival blood vessel rupture and rarely orbital subperiosteal haematoma formation, which can cause increased intraocular pressure and optic nerve compression requiring urgent needle aspiration or orbitotomy.^40^^,^^41^ Prevention involves the diver regularly exhaling through their nostrils, increasing the pressure within the mask and ensuring the volume remains constant.^42^ Diving has also been associated with arterial air embolism,^43^ which can be treated with hyperbaric oxygen therapy.^44^

Mountaineers, skiers and beachgoers are at risk of ultraviolet (UV) keratitis. UV keratitis, also known as snow blindness, is a photochemical injury secondary to the toxic effects of high-dose UV radiation. UV keratitis presents with ocular pain, tearing, conjunctival chemosis, blepharospasm and deterioration of vision. It typically occurs several hours after exposure and lasts up to 3 days.^45^

Solar retinopathy can occur due to unprotected gazing at the sun, especially during activities such as eclipse tourism or at higher altitudes. This can result in acute foveal injury and outer retinal defects.^46^ Using appropriate eye protection is crucial to prevent such issues. Travellers should use specialized solar filters or eclipse glasses that are certified and meet safety standards to ensure adequate protection. Sunglasses with sidepieces or goggles with polarized or photochromic lenses can also be effective in preventing common eye problems associated with these activities.^47^ The increased sun exposure associated with travel can lead to cataract formation.^48^

### Travel-related eye infections

Travellers are at a higher risk of acquiring an infection when they are unfamiliar with local destination hygiene conditions and climatic factors. Eye infections may be bacterial, viral, fungal or protozoan. An understanding of this condition is key for travel health practitioners as these infections are often preventable and treatable. One of the leading causes of blindness in the world is keratitis (Table 2). The occurrence of infectious keratitis is higher in developing countries with an incidence of up to 799 per 100 000 person-years in one landmark study.^49^ The type of organism varies depending on the geographic location. Fungal keratitis, for instance, is concentrated in Southern Asia, with an estimated global annual incidence of over a million.^50^ Risk factors for ocular infections include existing contact lens wear, ocular surface disease, eyelid disease, systemic disease and trauma, including post-ocular surgery.^51^ The signs and symptoms depend on the type of organisms, extent of infection and existing ocular state but in general patients may experience pain, photophobia and decreased vision. Keratitis is treated based on risk factors and local antimicrobial guidelines. Treatment involves the use of antimicrobial agents, cycloplegic eye drops, corticosteroids and corneal scrapings for culture and sensitivities. Close monitoring is essential to ensure effective treatment.

**Table 1:** Recommendations for travellers with visual impairment

Pre-travel	
Plan ahead	Contact airlines, hotels and attractions in advance to confirm accessibility and accommodations for people with visual impairment.
Documentation	Obtain necessary documentation such as a medical note or disability certificate to request special assistance and accommodations.
Pack	Pack essential equipment such as a white cane, magnifier and any other aids.
Communicate with airlines	Notify airlines in advance of any special needs or accommodations, such as pre-boarding, seating or assistance navigating the airport.
**During travel**	
Airport navigation	Request assistance navigating the airport, including security checkpoints and boarding the plane.
Accommodation	Book accommodations that have braille signage and large print material
Ground transportation	Consider the transportation options and make appropriate. Arrangements, such as utilizing taxis or public transportation.
Attraction	Enhance accessibility by using guided tours and consider utilizing assistive technologies like audio guides.

**Table 2:** Common causes of keratitis in travellers

Type of Pathogen	Causes
Bacteria	*Staphylococcus aureus*, *Staphylococcus epidermidis*, *Pseudomonas aeruginosa*, *Streptococcus pneumoniae*
Virus	Adenovirus, Herpes simplex virus, Varicella-zoster virus
Fungal	Fusarium, Aspergillus, Candida
Protozoan	Acanthamoeba

With the ongoing COVID-19 pandemic, there is a risk of travellers being infected with SARS-CoV-2. COVID-19 is well documented to cause a prothrombotic state by increasing inflammation, endothelial disruption and complement activation.^52^ This resulted in a high incidence of pulmonary embolism and deep vein thrombosis in patients infected with SARS-CoV-2.^53^ Equivalent findings in the eye would be a retinal vein or artery occlusion, which typically affects older individuals but can also occur in younger people who had the virus.^54^ Similar underlying mechanisms are proposed for ischaemic optic neuropathy secondary to disruption of the arterial blood supply.^55^ There are many other conditions in the literature that link COVID-19 with ocular conditions such as acute macular neuroretinopathy, central serous chorioretinopathy and even fungal endogenous endophthalmitis.^56–58^ Larger studies are needed to further characterize their relationship.

**Figure 1:**
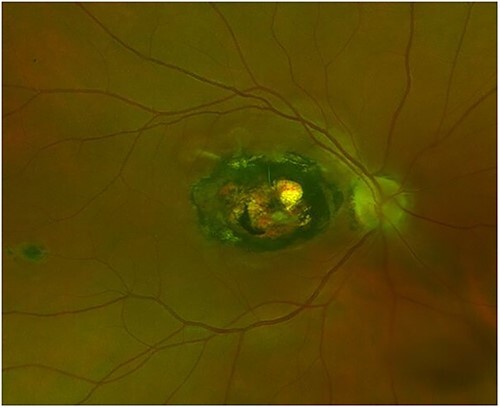
Ocular toxoplasmosis. Optos image of right fundus showing an established macular scar from toxoplasma. (Photo courtesy of Dr Tomas Burke)

Parasites are a common source of ocular inflammation, especially in visitors to tropical and subtropical regions. Toxoplasmosis is one of the most common parasitic ocular infections causing retinochoroiditis (Figure 1). It can be contracted by consuming undercooked meat or shellfish. In Brazil, antibodies to *Toxoplasma* have been demonstrated in up to 80% of the population.^59^ Moreover, the prevailing *Toxoplasma* genotype in Central and South America causes a more severe inflammatory response.^59^ There is a considerable risk of recurrence after an episode of necrotizing retinitis, which may require long-term monitoring.

**Figure 2:**
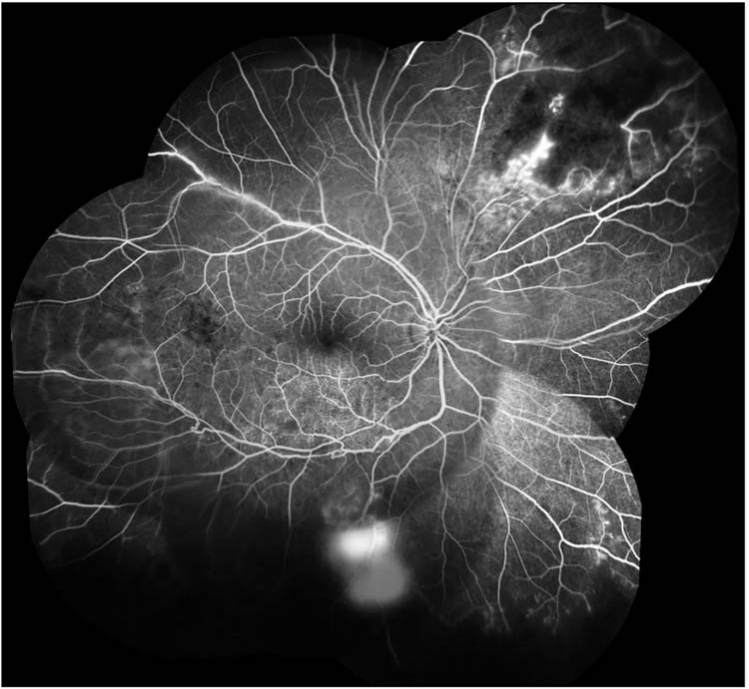
Tubercular retinal arteritis. Fundus fluorescein angiogram of the right eye showing multiple area of retinal ischaemia from vasculitis (Photo courtesy of Dr Deirdre Townley)

Malaria is another common parasitic infection that can affect the eyes, particularly in Sub-Saharan Africa. The pathogenesis of malaria retinopathy is like that of cerebral malaria, with vessel occlusion and ischaemia.^60^ While there is no known treatment for malaria retinopathy, effective chemoprophylaxis is available. One such prophylactic agent is hydroxychloroquine, which can cause retinopathy, although this is rare with short-term use.^61^ Travellers should also be advised to employ protective measures including applying insect repellent to their skin, minimizing skin exposure and avoiding forested and still water environments. Figure 2 illustrates a case of retinal arteritis occurring in a patient with tuberculosis (Figure 2).

Other rarer parasites that can cause posterior uveitis include *Cysticercus*, *Onchocerca* and *Gnathostoma*. While uncommon in Western Europe, they are endemic in some countries. The signs and symptoms vary between organisms, and management includes the use of anti-helminthic drugs and surgery in selected cases.

### Pre-existing eye conditions

For patients with pre-existing ocular conditions, it is recommended to pack sufficient medication in their carry-on bag in their original, labelled containers. While many countries allow a month’s supply of medicine, a prescription from the patient’s physician is often required. To ensure authenticity, it is best to use medication from the patient’s home country, especially in low- and middle-income countries where counterfeit drugs are more common.^62^^,^^63^ Environmental conditions such as humidity, temperature, wind, dust and pollen can affect ocular surface conditions like dry eyes syndrome, blepharitis and allergic conjunctivitis.^64^ If necessary, patients should consider increasing medication frequency to maintain control of the disease. Some ocular conditions, such as wet age-related macular degeneration, retinal vein occlusion and diabetic macular oedema, require treatment at regular intervals. Patients on active treatment should discuss their travel plans with their ophthalmologist to determine if they can connect them with a healthcare professional at their destination if their travel duration exceeds their medication interval.

### Contact lens use in travellers

While generally safe, contact lens wearers face an increased risk of complications during travel, including discomfort and infections. This risk is exacerbated by environmental factors and the challenge of maintaining good hygiene while on the go in an unfamiliar area, which is further compounded by limited access to ophthalmic care. These factors can lead to delayed treatment and worse outcomes for contact lens-related issues.

**Figure 3:**
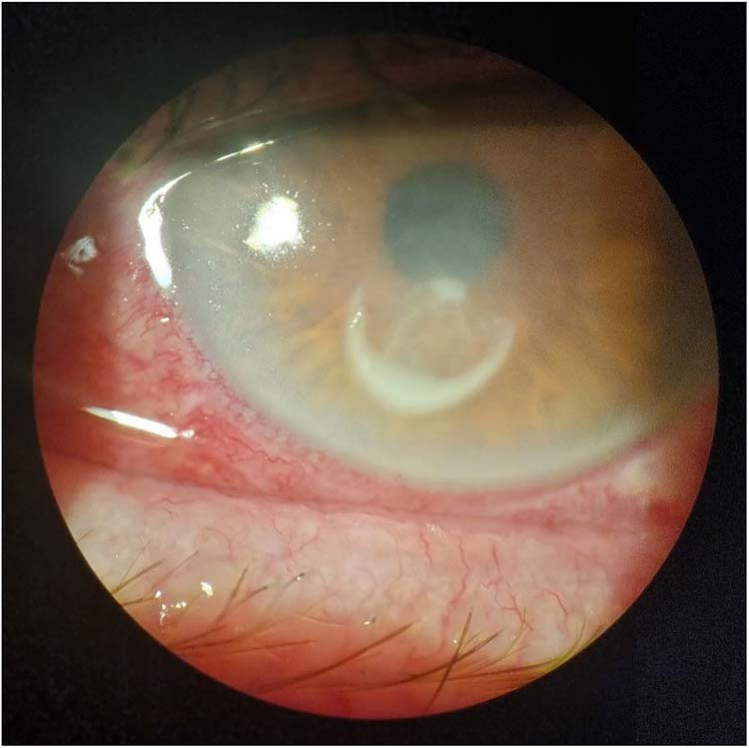
Contact lens-associated keratitis showing injected conjunctiva with ring-shaped corneal infiltrate (Photo courtesy of Dr Jay Jun Lee)

Risk factors for contact lens-related ulcers are overnight contact lens wear, long duration of continuous wear, lower socio-economic classes, smoking, dry eyes and poor hygiene.^65^ Compared with microbial keratitis without contact lens wear, the disease in contact lens wearers is more common, has a lower morbidity and is more often caused by *Pseudomonas aeruginosa* and *Acanthamoeba* species.^66^ Failing to wash and dry hands prior to handling lenses and poor hygiene with lens storage cases have been identified as key risk factors in microbial keratitis,^66^ which can be exacerbated by travellers having poor access to clean water and contact lens supplies. Dry air, dust, wind and cold temperatures can lead to contact lens discomfort and predispose the wearer to epithelial damage and secondary corneal ulceration. Daily disposable contact lenses have been shown to significantly reduce the relative risk of microbial keratitis compared with planned replacement soft contact lenses and rigid gas permeable contact lenses. Risk of microbial keratitis is up to 5.4 times higher with the use of extended wear contact lenses.^67^ Contaminated water exposure increases the risk of microbial keratitis in contact lens wearers (Figure 3), in particular *Acanthamoeba* keratitis.^68^ Therefore, recommendations should be made for daily contact lens wear over monthly or extended wear lenses and advice against swimming, showering or sleeping in contact lenses should be provided to all potential travellers. Prescription swimming goggles should also be considered.

Travel health professionals should assist individuals in evaluating the risks associated with contact lens wear and care in remote wilderness areas during pre-travel consultations. This includes considering the potential for infection due to lens contamination from touching, particularly for daily disposables, versus the risk of corneal erosion when using extended-wear lenses.^69^ It is essential for healthcare providers and travel agencies to remind contact lens wearers who plan to venture into remote areas to carefully review their lens wear and care routine to adapt to challenging conditions.

### Teleophthalmology

Teleophthalmology refers to the use of telecommunication technologies such as video conferencing, digital imaging and remote monitoring to provide eye care services to patients in remote or underserved areas with limited access to eye care. Well-established teleophthalmology programs in Canada, India and Western Australia have allowed improved eye care delivery through swift assessment, improved access and reduced travel expenses and time.^70–73^ However, previous programs have focused on chronic diseases such as glaucoma and diabetic retinopathy. Additional research may be required for successful implementation of teleophthalmology programs in acute ophthalmic presentations. The COVID-19 pandemic has accelerated the adoption and evolution of ophthalmology virtual clinics. This shift towards virtual eye care consultations has paved the way for future developments in teleophthalmology, making it an increasingly viable and convenient solution for patients seeking eye care services.^74^^,^^75^

### Ophthalmic tourism

In 2017, global spending on medical tourism reached $11 billion, increasing from $2.2 billion in 2000.^76^ This trend is largely driven by patients from developed countries seeking more affordable medical treatment in developing countries. Ophthalmic procedures such as laser assisted in situ keratomileusis (LASIK) are particularly popular among medical tourists, with treatment costs starting at $360 for both eyes in India, compared to around $2000 in the United States.^77^^,^^78^ While accessibility, availability and quality of care are factors that influence the decision to seek medical treatment abroad, affordability remains a key consideration. Patients should be aware, however, that medical training standards and laws governing medical liability may vary between countries, and they may not have access to long-term follow-up care. In addition, any complications that arise during or after treatment may increase the burden on their home country’s healthcare system.

### Limitations of review

While this review is one of the first to focus on eye health and international travel, it is subjected to several limitations, including the fact that eye disease in the context of space travel and high-altitude travel are under-researched areas. Additionally, reliance on English language publications may have introduced selection bias, and the use of two databases may have caused important literature to have been overlooked.

### Recommendations for further research

Despite the importance of eye health in travel medicine, there is a lack of adequate discussion on ocular health in related journals. To address this knowledge gap, further research is needed in assessing the safety of travelling with visual impairment and developing supportive strategies for affected individuals. Identifying effective prophylactic measures and optimal treatment options for ocular diseases that occur during travel is also necessary. Additionally, understanding the psychological impact of ocular diseases on travellers and providing effective support is essential. Investigating the correlation between the duration of space travel and the severity of ocular disease is an important area to explore. Finally, creating evidence-based guidelines for the prevention, diagnosis and treatment of ocular diseases in travellers is critical. By conducting research in these areas, we can improve our understanding of ocular health in travellers, leading to better care and support for affected individuals.

## Conclusion

Travel medicine practitioners should have a comprehensive understanding of the major ocular risks associated with overseas travel, including eye infections, eye injuries and solar eye damage. Further research in this area can enhance overall wellness and alleviate the burden of ocular diseases on travellers. Evidence-based guidelines based on research can also improve the quality of care and prevent long-term vision problems.
